# Outcome after Radiotherapy for Vestibular Schwannomas (VS)—Differences in Tumor Control, Symptoms and Quality of Life after Radiotherapy with Photon versus Proton Therapy

**DOI:** 10.3390/cancers14081916

**Published:** 2022-04-10

**Authors:** Maike Küchler, Rami A. El Shafie, Sebastian Adeberg, Klaus Herfarth, Laila König, Kristin Lang, Juliane Hörner-Rieber, Peter Karl Plinkert, Wolfgang Wick, Felix Sahm, Simon David Sprengel, Jürgen Debus, Denise Bernhardt

**Affiliations:** 1 Department of Radiation Oncology, University Hospital of Heidelberg, INF 400, 69120 Heidelberg, Germany; maikekuechler@gmail.de (M.K.); rami.elshafie@med.uni-goettingen.de (R.A.E.S.); sebastian.adeberg@med.uni-heidelberg.de (S.A.); klaus.herfarth@med.uni-heidelberg.de (K.H.); laila.koenig@med.uni-heidelberg.de (L.K.); kristin.lang@med.uni-heidelberg.de (K.L.); simon.sprengel@med.uni-heidelberg.de (S.D.S.); juergen.debus@med.uni-heidelberg.de (J.D.); 2 Department of Radiation Oncology, University Medical Center Göttingen, Robert-Koch-Straße 40, 37075 Göttingen, Germany; 3 Deutsches Konsortium für Translationale Krebsforschung (DKTK), Partner Site Heidelberg, INF 280, 69120 Heidelberg, Germany; felix.sahm@med.uni-heidelberg.de; 4 Heidelberg Institute of Radiation Oncology (HIRO), INF 400, 69120 Heidelberg, Germany; juliane.hoerner-rieber@med.uni-heidelberg.de; 5 Clinical Cooperation Unit Radiation Oncology, German Cancer Research Center (DKFZ), INF 280, 69120 Heidelberg, Germany; 6 National Center for Tumor Diseases (NCT), INF 460, 69120 Heidelberg, Germany; 7 Heidelberg Ion Beam Therapy Center (HIT), INF 450, 69120 Heidelberg, Germany; 8 Department of Neurology, University Hospital of Heidelberg, INF 400, 69120 Heidelberg, Germany; wolfgang.wick@med.uni-heidelberg.de; 9 Department of Otolaryngology, University Hospital of Heidelberg, INF 400, 69120 Heidelberg, Germany; peter.plinkert@med.uni-heidelberg.de; 10 Department of Neuropathology, Institute of Pathology, University Hospital Heidelberg, INF 224, 69120 Heidelberg, Germany; 11 Department of Radiation Oncology, School of Medicine, Klinikum rechts der Isar, Technical University of Munich (TUM), 80333 Munich, Germany; 12 Department of Radiation Sciences (DRS), Institute of Radiation Medicine (IRM), Ingolstädter Landstraße 1, 85764 Neuherberg, Germany; 13 Deutsches Konsortium für Translationale Krebsforschung (DKTK), Partner Site Munich, Pettenkoferstraße 8a, 80336 München, Germany

**Keywords:** vestibular schwannomas, stereotactic radiotherapy, proton therapy, clinical outcome, tumor control, hearing preservation, quality of life

## Abstract

**Simple Summary:**

The standard of care for radiotherapy of symptomatic or progressive vestibular schwannomas (VS) is photon beam single-dose radiosurgery (applying 1 × 12 Gy) or (hypo)fractionated radiotherapy (applying 3 × 6 Gy up to 32 × 1.8 Gy). Only few centers also enable irradiation with protons. Proton therapy offers unique physical properties whereby healthy tissue around the tumor can be protected although a very high dose is applied to the target lesion. In patients with benign brain tumors such as vestibular schwannomas reduction of treatment-related side effects is very important. Few data comparing photon vs. proton beam radiotherapy for patients with VS are available. Thus, a detailed evaluation of differences in tumor control, symptoms and quality of life in patients with VS after photon beam vs. proton beam radiotherapy is needed.

**Abstract:**

Background: To evaluate differences in local tumor control (LC), symptoms and quality of life (QOL) of 261 patients with VS after stereotactic radiosurgery/hypofractionated stereotactic radiotherapy (SRS/HFSRT) vs. fractionated radiotherapy (FRT) vs. fractionated proton therapy (FPT) were studied. Methods: For SRS/HFSRT (*n* = 149), the median fraction dose applied was 12 Gy. For FRT (*n* = 87) and FPT (*n* = 25), the median cumulative doses applied were 57.6 Gy and 54 Gy (RBE), respectively. FRT and FPT used single median doses of 1.8 Gy/Gy (RBE). Median follow-up was 38 months. We investigated dosimetry for organs at risk and analyzed toxicity and QOL by sending out a questionnaire. Results: LC was 99.5% at 12 months after RT with no statistical difference between treatment groups (*p* = 0.19). LC was significantly lower in NF2 patients (*p* = 0.004) and in patients with higher tumor extension grade (*p* = 0.039). The hearing preservation rate was 97% at 12 months after RT with no statistical difference between treatment groups (p = 0.31). Facial and trigeminal nerve affection after RT occurred as mild symptoms with highest toxicity rate in FPT patients. Conclusion: SRS/HFSRT, FRT and FPT for VS show similar overall clinical and functional outcomes. Cranial nerve impairment rates vary, potentially due to selection bias with larger VS in the FRT and FPT group.

## 1. Introduction

Vestibular schwannoma (VS, acoustic neuroma) is a usually benign developing tumor derived from the nerve sheath of the eighth cranial nerve. Incidence rates found modern population-based studies ranging between 1.1–1.9 per 100,000. The majority of tumors occur unilaterally and sporadically. Furthermore, neurofibromatosis type 2 (NF2) is associated with bilateral tumors. Standard management of VS includes observation, (micro)surgery, and radiotherapy (RT). For smaller lesions, stereotactic radiosurgery (SRS) as a non-invasive approach for definitive treatment for small to medium-sized lesions leads to similar local control and potentially better functional outcome and quality of life (QOL) compared to surgery. In the case of large and irregularly shaped lesions, surgery is typically preferred, especially if the tumor reaches the brain stem. However, fractionated RT (FRT) can be used when surgery is not feasible. Previous data shows that SRS and FRT with modern techniques can achieve similar results in terms of local control and hearing function, although FRT allows treating larger lesions in closer proximity to the brain stem [1]. Fractionated proton radiotherapy (FPT) for VS achieves high tumor control rates, equivalent to photon irradiation. FPT enables a reduction of dose to normal tissue and thus the potential of reducing treatment-associated toxicities. FPT is mostly used for larger VS. Reported cranial nerve preservation rates are inconclusive, also due to a selection bias with a high percentage of patients with larger tumors or clinical symptoms prior to treatment. The primary scientific aim of this retrospective exploratory analysis is a detailed evaluation of differences in tumor control, symptoms, and quality of life in patients with VS after SRS/HFSRT, FRT and FPT. We aim to give a detailed investigation of VS patients treated with proton beam radiotherapy because few data are currently available.

## 2. Materials and Methods

### 2.1. Patient Characteristics

Between 2007 and 2019, 261 patients with VS were treated at the Department of Radiation Oncology at the Heidelberg University Hospital, Germany, or the Heidelberg Heavy Ion Therapy Center (HIT). We defined the following inclusion criteria: patients (>18 years) who received stereotactic radiotherapy either with photon or proton beam at the Department of Radiation Oncology at the Heidelberg University Hospital, or the Heidelberg Heavy Ion Therapy Center (HIT). Patients who underwent neurosurgery prior to their RT and patients with neurofibromatosis type 2 were also included. Patients without VS, patients with VS without receiving stereotactic radiotherapy or with an age under 18 years or without informed consent were excluded from the study.

Patient data were collected retrospectively by using information documented in the patient history and from clinical assessments during follow-up. Furthermore, an adapted questionnaire was sent to all 261 patients. This questionnaire is called Heidelberg SYQOL Inventory: questionnaire on symptom control, outcome, and quality of life (QOL) for self-reported outcome. It was initially developed by Combs et al. [2] and applied in a large cohort treated by SRS and FRT. This questionnaire was completed by 168 patients (64.4%). The Heidelberg SYQOL Inventory explicitly refers to typical symptoms, side effects, long-term outcome, and quality of life of patients with VS prior to RT and after treatment. A detailed survey about hearing ability after RT was also included. We asked for hearing function with the ipsilateral and contralateral ear in situations with different levels of background noises as well as differences in hearing ability with the use of a hearing aid. Overall, the Heidelberg SYQOL Inventory differs from conventional instruments and is particularly suitable for recording symptoms and QOL of patients with VS. 

Of all 261 patients, 149 patients (57.1%) were treated with single-fraction SRS (*n* = 120) or hypofractionated stereotactic radiotherapy (HFSRT, *n* = 29) in 3 fractions, 87 patients (33.3%) were treated with FRT and 25 (9.6%) with FPT. Of the total number patients included, 130 patients were male (49.8%), and 131 were female (50.2%). Median age was 59 years (IQR: 48–71 years). Median tumor volume was 0.8 ccm (IQR: 0.4–1.6 ccm). For 205 patients (78.5%), RT was performed as primary treatment for VS. Thus, 56 patients (21.5%) have already had neurosurgery prior to RT. For these patients, median time from surgery to subsequent RT was 43 months (IQR: 24–81 months). Patient characteristics are summarized in Table 1. 

Follow-up included clinical examination and radiological scan with MRI. Tumor progression was defined according to the RECIST criteria. Hearing ability was scored according to the Gardner–Robertson Classification (GR) by using clinical information, patient questionnaire and standardized audiograms from otolaryngologists, if available. GR Class I and II were defined as useful hearing and Class III–V as non-useful hearing. Hearing deterioration was defined as loss of useful hearing. If patients reported loss of useful hearing, we used time of first audiometry, which marks Gardner Robertson Class III–V. Facial nerve impairment was assessed using the House–Brackmann score (HB).

All data were collected in accordance with the World Medical Association Declaration of Helsinki. The local ethics committee of the Medical Faculty of the University of Heidelberg approved the study with vote number S-832/2018.

### 2.2. Treatment Planning

Patients with VS were mostly treated with SRS/HFSRT or FRT by using photon beam for standard of care. Treatment decision for SRS or HFSRT is based on tumor size, proximity to the brain stem and patients’ characteristics. Patients with larger VS and therefore larger CTV were more likely to receive HFSRT to allow normal tissues to recover between fractions. The decision for an irradiation with proton beam was based on physicians’ choice regarding tumor volume, patient characteristics and patient history. Furthermore, the decision depends on the patients’ health insurance and the assumption of treatment costs. FRT was often used for patients with larger VS, multiple surgeries prior to RT or tumor progression. For all cranial RT, an individual thermoplastic head fixation mask was fitted for each patient. Treatment by SRS/HFSRT was delivered using Accuray’s CyberKnife M6 system with fixed cone collimators, whereas FRT was delivered using an adapted linear accelerator. FPT was delivered at the Heidelberg Ion Therapy Center (HIT) using active raster-scanning for beam delivery. Treatment planning was based on high-resolution computed tomography (CT) and magnetic resonance imaging (MRI). Standardized imaging protocols were used for all patients, complying to the following specifications: CT scan was acquired with 1–3 mm slice thickness. MRI was thoroughly co-registered and contained a contrast-enhanced, T1-weighted, three-dimensional sequence with multiplanar reconstruction and a slice thickness of ≤1 mm for SRS/HFSRT and at least ≤ 3 mm for FRT. Additionally, a high-resolution T2-weighted sequence was required for the identification of neural structures and organs at risk (OAR). Gross tumor volume (GTV) consisted of all contrasted tissue in the T1-weighted MRI. A safety margin of 1 mm was added to the GTV by isotropic expansion to create the planning target volume (PTV) for SRS/HFSRT and FRT. PTV margin for FPT could be expanded up to 3 mm at the discretion of the responsible dosimetrist. Dose prescription and OAR dosimetry adhered to current guidelines, as well as recommendations set down by QUANTEC [3,4] and Emami et al. [5]. Details regarding doses to the PTV and OAR are listed in the Results section.

### 2.3. Statistical Analysis

The study is based on an initially created study protocol, which also contains specifications for the statistical analysis. For baseline analyses, descriptive statistics are used, continuous variables are given as means (standard deviation, SD) and/or median (interquartile range (IQR) and range, as appropriate) and categorical variables as absolute and relative frequencies. We used the Kaplan–Meier method to calculate rates for local control and hearing preservation. Intergroup differences were analyzed by using log-rank test and Cox regression with univariate and multivariate analysis. Correlation between risk factors and toxicity was assessed using the Chi square test and the Odds ratio. Since this was a retrospective exploratory data analysis, p-values are of descriptive nature. A descriptive *p*-value of <0.05 was considered as statistically significant and 95% confidence intervals (95%-CI) are provided together with the respective p-values. All statistical analysis was carried out using the IBM SPSS Statistics version 26 (IBM, Armonk, NY, USA).

## 3. Results

### 3.1. Tumor Volume and Tumor Expansion

Of all patients (*n* = 261), median tumor volume was 0.8 ccm (IQR: 0.4–1.6 ccm). For SRS/HFSRT, FRT and FPT, median tumor volume was 0.7 ccm (IQR: 0.3–1.1 ccm), 1.6 ccm (IQR: 1.0–2.6 ccm) and 3.9 ccm (IQR: 2.3–6.2 ccm), respectively. We used the Hannover Vestibular Schwannoma grading scale for tumor expansion and results are shown in Table 2. Furthermore, we subclassified patients in two groups regarding VS with and without brain stem contact. Hannover grade T1–T3a was classified as VS without brain stem contact (equivalent to Koos classification grade I–II) and Hannover grade T3b–T4b represented VS with brain stem contact or compression (equivalent to Koos classification grade III–IV). We found that SRS/HFSRT patients had significantly lower tumor volume and tumor expansion grade than FRT and FPT (Table 1 and Table 2).

### 3.2. Dosimetry

All patients treated with SRS/HFSRT (*n* = 149) received a median dose of 12 Gy. The median prescription isodose line was 80% (68–80%). Of these patients, we treated 120 (80.5%) with single-fraction SRS using a median margin dose of 12 Gy (IQR: 12–12 Gy). Twenty-nine patients (19.5%) treated with hypofractionated stereotactic radiotherapy (HFSRT) were all trested in three fractions with single doses of 6 Gy. In the FRT group, the median prescribed total dose was 57.6 Gy (IQR: 57.0–57.6 Gy), which was applied in single doses of 1.8 Gy (n = 86) or 2.0 Gy (*n* = 1). Therefore, these patients underwent a median of 32 fractions. In patients receiving FPT, median dose was 54.0 Gy (IQR: 54.0–57.6 Gy) using single median doses of 1.8 Gy (RBE) (IQR: 1.8–2.0 Gy (RBE)) with median fractionation of 30 (IQR: 27–32).

We analyzed dosimetric information of organs at risk, which is shown in Table 3. Dosimetry of cochlea, labyrinth and facial nerve was only available for SRS/HFSRT and FPT, but not for FRT. We documented the highest single doses in organs at risk in patients with single-dose SRS (*n* = 120). These patients received a median mean dose to the cochlea, labyrinth, facial nerve, and brain stem of 7.2 Gy, 5.6 Gy, 5.2 Gy and 1.6 Gy, respectively. Although patients with FRT and FPT received higher total doses in organs at risk, due to fractionation they had relevantly lower single doses. For FPT, the median mean total doses to the cochlea, labyrinth, facial nerve, and brain stem were 50.7 Gy (RBE), 43.6 Gy (RBE), 43.5 Gy (RBE), and 11.4 Gy (RBE), respectively.

### 3.3. Local Control

**Figure 1:**
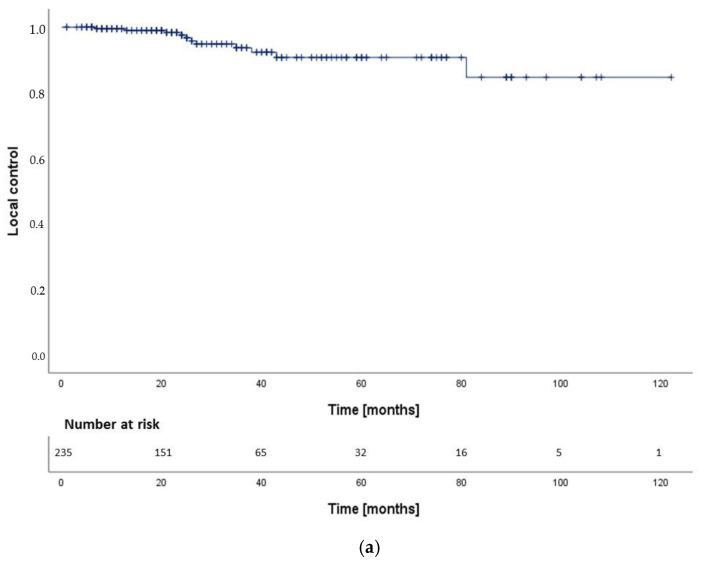

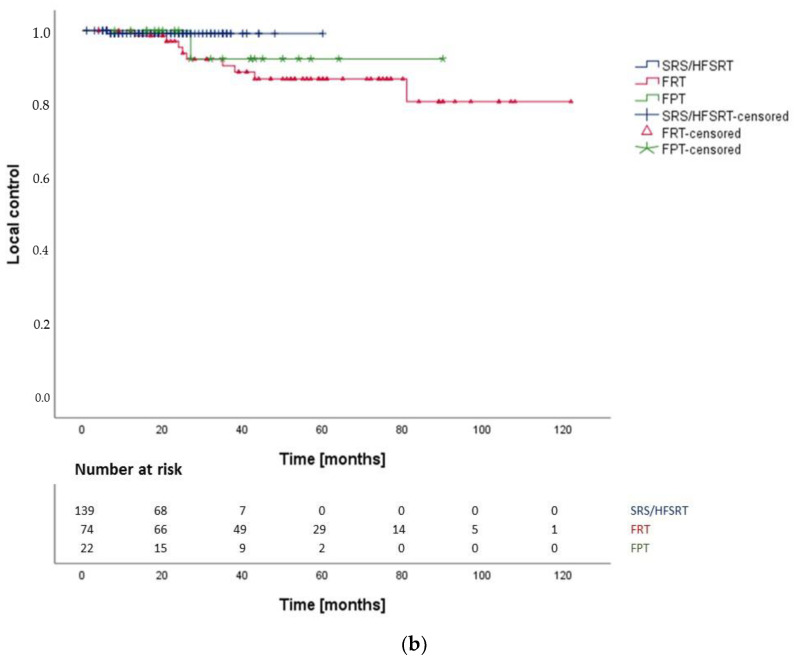
(**a**) LC after RT in 235 patients; (**b**) LC after SRS/HFSRT (*n* = 139) vs. FRT (*n* = 74) vs. FPT (*n* = 22) (*p* = 0.19); SRS-HFSRT/FRT (HR: 0.2, 95%-CI: 0.0–1.5), SRS-HFSRT/FPT (HR: 0.3, 95%-CI: 0.0–4.7) and FRT/FPT (HR: 1.8, 95%-CI: 0.2–14.3).

**Figure 2:**
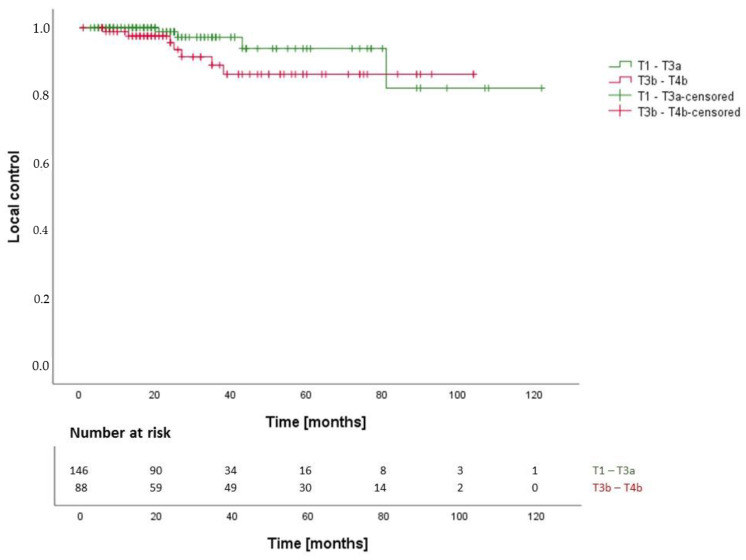
Local control in VS with lower (T1–T3a) and higher (T3b–T4b) tumor extension grade.

After a median follow-up time of 38 months (IQR: 21–78 months), actuarial local control (LC) rates were 99.5% at 12 months, 93.7% at 36 months and 90.8% at 72 months after RT (Figure 1a). We found no statistically significant difference between SRS/HFSRT and FRT and FPT (*p* = 0.19, 95%-CI: 104.0–117.9 months) regarding LC (Figure 1b). We calculated the following hazard ratios (HR) for LC: SRS-HFSRT/FRT (HR: 0.2, 95%-CI: 0.0–1.5), SRS-HFSRT/FPT (HR: 0.3, 95%-CI: 0.0–4.4) and FRT/FPT (HR: 1.8, 95%-CI: 0.2–14.3). Overall, tumor progression was observed in 11 patients (4,7%). Local control was higher in patients with sporadic VS than in patients with NF2 (*p* = 0.004, 95%-CI (NF2): 24.8–106.2 months) with an HR (sporadic/NF2) of 0.2 (95%-CI: 0.0–0.9). Moreover, we reported significantly higher local control in patients with lower tumor extension grade using the Hannover VS grading scale (*p* = 0.039, 95%-CI: 85.9–100.8 months) (Figure 2). Of all 11 patients with tumor progression, 7 patients (63.6%) had VS with brain stem contact or compression (T3b-T4b). Regarding differences in distribution of tumor extension between the treatment groups (Table 1 and Table 2), 10 out of 11 patients with progression received FRT (*n* = 9) or FPT (*n* = 1) and one patient developed tumor progression after SRS. This patient had a large VS with brain stem contact (T3b). We found no statistically significant difference between pre-operated and non-operated patients regarding local control (*p* = 0.98, HR: 1.0, 95%-CI: 0.3–3.7).

### 3.4. Hearing Preservation

**Figure 3:**
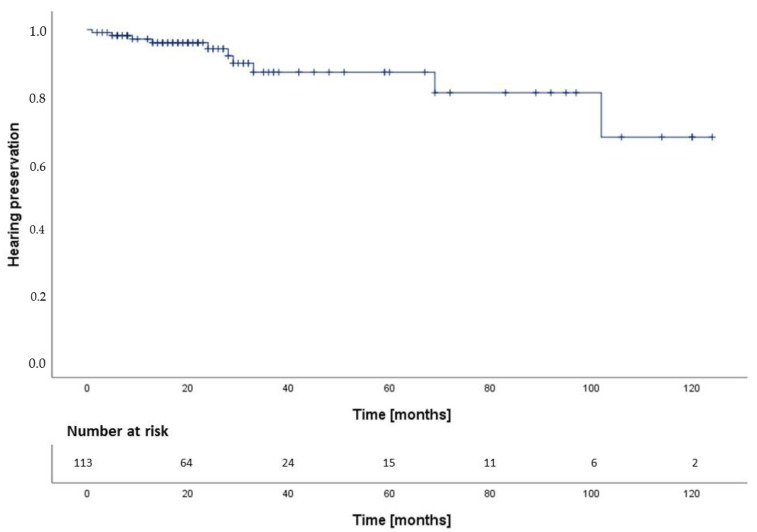
Overall hearing preservation after RT.

**Figure 4:**
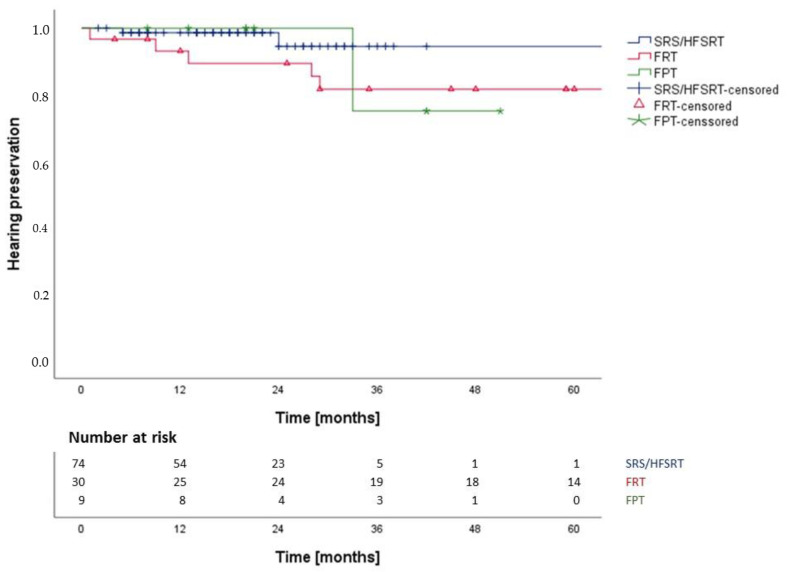
Hearing preservation after SRS/HFSRT vs. FRT vs. FPT (*p* = 0.31); SRS-HFSRT/FRT (HR: 0.3, 95%-CI: 0.1–1.4), SRS-HFSRT/FPT (HR: 0.4, 95%-CI: 0.0–5.3) and FRT/FPT (HR: 1.2, 95%-CI: 0.1–10.5).

Useful hearing was classified as Gardner-Robertson-Class I and II, non-useful hearing as Class III–V. In patients with useful hearing before treatment, actuarial hearing preservation rate was 97.1% at 12 months, 94.2% at 24 months and 87.1% at 60 months after RT (Figure 3). There was no statistical difference between SRS/HFSRT and FRT and FPT (*p* = 0.31, 95%-CI: 92.9–116.1 months) (Figure 4). We calculated the following hazard ratios: SRS-HFSRT/FRT (HR: 0.3, 95%-CI: 0.1–1.4), SRS-HFSRT/FPT (HR: 0.4, 95%-CI: 0.0–5.3) and FRT/FPT (HR: 1.2, 95%-CI: 0.1–10.5). Excluding all patients without prior useful hearing (*n* = 78, 35.6%), we therefore found hearing deterioration in 17.8% of patients treated with FRT, in 16.7% of patients treated with FPT and in 3.6% of patients treated with SRS/HFSRT. In these patients with hearing deterioration after SRS/HFSRT (*n* = 3), the median maximum dose to the cochlea was 11.8 Gy (IQR: 11.1–17.7 Gy) and the median mean dose to the cochlea was 7.1 Gy (IQR: 6.1–12.7 Gy). In patients with preserved useful hearing after SRS/HFSRT (*n* = 81), the median maximum dose to the cochlea was 11.7 Gy (IQR. 9.8–12.9 Gy) and the median mean dose to the cochlea was 6.6 Gy (IQR: 5.7–8.1 Gy). This difference between the doses to the cochlea in SRS/HFSRT patients with and without hearing deterioration was not statistically significant (*p* = 0.56). Of the SRS/HFSRT patients with hearing deterioration, 33.3% had VS with brain stem compression (T4a). Of all patients with preserved useful hearing after SRS/HFSRT, most had smaller VS with no brain stem contact (85.2%). Of all patients with prior useful hearing and tumor progression after RT (*n* = 4), only one suffered from hearing deterioration. In patients with NF2 and audiological follow up (n = 4), no increased risk of hearing deterioration was reported (*p* = 0.60, HR (sporadic/NF2): 0.6, 95%-CI: 0.1–4.8) We found no statistically significant difference in hearing preservation between pre-operated and non-operated patients (*p* = 0.44, HR: 0.6, 95%-CI: 0.1–2.6).

Most patients who completed our questionnaire indicated problems with directional hearing (60.1%) and communication in noisy surroundings (71.4%). Using a hearing aid relevantly improved directional hearing in 17.3% of patients and communication in 12.4% of patients.

### 3.5. Tinnitus

Tinnitus was present in 71.4% in patients prior to SRS/HFSRT, in 70.1% in patients prior to FRT and in 63.6% in patients prior to FPT. Improvement of tinnitus was documented in 6.1% after SRS/HFSRT and in 7.8% after FRT. Development of new tinnitus appeared in 4.1% of the cases after SRS/HFSRT and in 2.6% after FRT. After FPT, no change in tinnitus was reported. Of all patients developing new tinnitus, none had an NF2.

### 3.6. Dizziness, Imbalance, Gait Uncertainty

Prior to RT, 80 patients reported dizziness (31.6%). After RT, 23.8% of these patients reported improved dizziness. Only 2.9% (*n* = 5) of patients at risk (no dizziness prior to RT, *n* = 173) developed new dizziness after RT. Two of them received SRS and a mean median dose of the labyrinth of 2.7 Gy. Median mean dose of the labyrinth in patients without new dizziness was 5.7 Gy. Three patients developed new dizziness after FRT, for whom no dosimetric information for the labyrinth was available. No patient with new dizziness received FPT and no one had an NF2. 

Imbalance was documented in 163 patients (65.2%) prior to RT. In 19.6% of these patients, imbalance was improved after RT, and 24.1% (*n* = 21) of patients at risk (*n* = 87) suffered from new imbalance after treatment, of whom one patient had an NF2. Eight patients with new imbalance received SRS and a mean median dose of the labyrinth of 5.7 Gy. Median mean dose of the labyrinth in patients without new imbalance was 6.1 Gy. Again, the reason for new dizziness in these patients after SRS was probably not the dose exposure of the labyrinth. Furthermore, we found new imbalance in two patients after HFSRT, similarly without any indication of dosimetric correlation. Three of nine patients without imbalance prior to FPT had new imbalance after FPT. These patients received a mean median dose of the labyrinth of 46.3 Gy (RBE). Median mean dose of the labyrinth in patients without new imbalance after FPT was 43.2 Gy (RBE). Thus, dosimetric correlation between applied dose in the labyrinth and development of new imbalance seems possible, though not very likely. Eight patients developed new imbalance after FRT, for whom no dosimetric information for the labyrinth was available. 

The presence of gait uncertainty prior to RT was 49.7% (*n* = 124). After RT, 25.8% of these patients reported improvement of gait uncertainty, and 15.0% of patients at risk (*n* = 127) developed new gait uncertainty after RT. We found that patients with NF2 had a higher risk for developing new gait uncertainty than patients with sporadic VS (OR 5.6, 95%-CI: 0.3–93.8); this difference was not statistically significant (*p* = 0.18). Eight patients suffered from new gait uncertainty after SRS/HFSRT. They received a median mean dose of the labyrinth of 11.5 Gy (SRS) and 19.6 Gy (HFSRT). Median mean dose in the labyrinth in patients without new gait uncertainty was 12.6 Gy (SRS) and 19.5 Gy (HFSRT). Thus, the reason for new gait uncertainty in these patients after SRS/HFSRT was probably not the dose exposure of the labyrinth. Six patients developed new gait uncertainty after FRT, for whom no dosimetric information for the labyrinth was available. Of FPT patients without gait uncertainty prior to RT (*n* = 12), 20.8% (*n* = 5) developed new gait uncertainty after FPT. These patients received a median mean dose of the labyrinth of 46.3 Gy (RBE). Median mean dose of the labyrinth in patients without new gait uncertainty after FPT was 42.2 Gy (RBE). 

### 3.7. Facial and Trigeminal Nerve Toxicity

Prior to RT, 44 patients suffered from facial nerve impairment. Thus, 216 patients (83.1%) were at risk for facial nerve toxicity. After RT, 15 of these 216 patients (6.9%) developed new facial nerve impairment, with a higher toxicity rate in patients receiving FPT (5/18, 27.8%). All these FPT patients had large VS with brain stem contact (20%) or even brain stem compression (80%). After SRS/HFSRT and FRT, facial nerve toxicity rates were 6.4% and 2.7%. In patients after SRS/HFSRT, we found lower risk for facial nerve impairment for those who received HFSRT (doses of 3x6 Gy) than in patients with SRS (single exposure of 12 Gy), (OR 0.6, 95%-CI: 0.1–5.3). This difference was not statistically significant (*p* = 0.66). Regarding House–Brackmann-Class (HB), an improvement of facial nerve function was reported in 6.5% of all patients, and in 87.7% there was no change in HB Class. Most patients suffering from new facial nerve impairment only had mild or temporary symptoms and switched from HB Class 1 to HB Class 2. Only one patient (0.4%) suffered from higher-grade facial nerve toxicity (HB Class 5) after single-exposure SRS with a maximum dose in the facial nerve of 13.9 Gy. This patient had already undergone multiple surgeries before RT due to a rapidly growing VS. On the date of RT, this VS was classified as grade T3a. We also found higher risk for facial nerve impairment after RT in patients with NF2 than in patients with sporadic VS (OR 2.9, 95%-CI: 0.3–26.2); this difference was not statistically significant (*p* = 0.42). NF2 patients with facial nerve affection had VS with brain stem compression (T4a).

Trigeminal nerve affection was reported in 48 patients before RT, and 204 patients (81.0%) were at risk for trigeminal nerve toxicity. Of these 204 patients, 22 (10.8%) developed new trigeminal nerve impairment after RT, with the highest toxicity rate in patients receiving FPT (3/15, 20.0%), from whom 2 patients (66.7%) had VS with brain stem compression (T4a). Lower toxicity rates were found in patients with SRS/HFSRT (10.2%) and with FRT (9.9%). In patients after SRS/HFSRT, we found lower risk for trigeminal nerve impairment for HFSRT than in patients with SRS (OR 0.4, 95%-CI: 0.1–3.4); this difference was not statistically significant (*p* = 0.40). All patients who suffered from new trigeminal nerve affection, mostly reported mild and temporary symptoms. Of all patients, even 5.6% had an improvement of developing nerve impairment after RT. Again, risk for developing new trigeminal nerve affection was higher in patients with NF2 than in patients with sporadic VS (OR 2.0, 95%-CI: 0.2–18.9, this difference was not statistically significant, *p* = 0.53) and was associated with large VS with brain stem compression (T4a).

### 3.8. Non-Specific Side Effects

Of all patients who answered the questionnaire (*n* = 168), few stated non-specific side effects after RT: temporary impaired vision (7.1%), temporary concentration or memory disorder (5.4%), speech disorder (4.2%), headache (3.0%), fatigue (2.4%), earache (1.2%). Impaired vision included blurred vision, diplopia, scotoma, and visual reduction. Visual symptoms only occurred in patients after SRS/HFSRT and after photon FRT, and they were higher represented in patients with NF2 than in patients with sporadic VS (OR 4.5, 95%-CI: 0.4–47.1); this difference was not statistically significant (*p* = 0.17).

### 3.9. Quality of Life (QOL) and General Condition

In the Heidelberg SYQOL Inventory questionnaire, we asked our treated patients whether QOL and general condition were improved, unchanged or decreased after RT compared to their QOL and general condition prior to RT. Overall QOL was improved in 29.9% of the patients, and it was unchanged in 50.9% after RT. Thus, 19.2% reported a decrease of QOL. After RT, overall general condition was improved in 31.7% of all patients, and it was unchanged in 50.3%. A reduction of general condition was only reported in 18.0% of patients. The reduction rate of QOL and of general condition was lower in the SRS/HFSRT group (both 15.6%) than in the FRT group (27.3%) and in the FPT group (25.0%). Patients with NF2 had a trend towards a higher risk for a reduction of QOL and of general condition than patients with sporadic VS (OR 1.5, 95%-CI: 0.2–15.0 and 1.6, 95%-CI: 0.2–15.7); the difference was not statistically significant (*p* = 0.73 and *p* = 0.70).

## 4. Discussion

In this study, we evaluated and compared clinical outcome and toxicity in 261 patients with VS treated with SRS/HFSRT, FRT and FPT. Our results indicate high tumor control rates after SRS/HFSRT, FRT and FPT for treatment of VS. We found actuarial local control rates of 99.5%, 93.7% and 90.8% at 1, 3 and 6 years after RT with no significant difference between the treatment groups. Statistically lower local control was present in patients with higher tumor extension grade. Moreover, we report excellent hearing preservation rates of 97%, 94% and 87% at 1, 2 and 5 years after RT. Between the three techniques, no significant difference in hearing preservation rates could be found. Facial nerve toxicity and trigeminal nerve affection were mostly reported as mild and temporary symptoms. However, FPT was associated with a higher rate of new facial nerve and trigeminal nerve impairment after RT; FPT patients all had large VS with brain stem contact or even compression. The reduction rate of QOL and of general condition was lower in the SRS/HFSRT group (both 15.6%) than in the FRT group (27.3%) and in the FPT group (25.0%), which indicates a lower QOL in patients with larger and potentially more symptomatic tumors.

Stereotactic radiotherapy has become a standard non-invasive treatment alternative to surgery, especially for small to medium-sized VS. Regarding local tumor control, hearing preservation, cranial nerve toxicity and QOL, RT has an equal or even more beneficial risk profile compared to surgery in smaller tumors. However, surgery is preferred for larger lesions with brain stem contact or for irregularly shaped lesions, and FRT can be used if surgery is not feasible. Previous data indicate that stereotactic radiotherapy can achieve similar results to surgery regarding local control and hearing preservation [6]. Moreover, it is known that RT enables lower treatment related cranial nerve toxicity than surgery [6,7,8].

Considering limited comparability due to different subgroup size and differences in clinical baseline characteristics, our analysis found roughly similar results in local control between SRS/HFSRT and FRT, which was also reported in previous data. LC for photon beam stereotactic RT is reported to be 86.2–100% [1,2,9,10,11,12,13]. A recent review investigating the outcome of large VS after RT showed that tumor control was achieved in 89%, 94% and 91% of patients respectively. NF2 patients remain a unique subgroup of VS patients with suspected lower rates of tumor control after radiation therapy. Comparable to previous reports, we found lower local control rates compared to non-NF2-patients, regardless of the treatment technique [14]. Odds ratios (ORs) of post- over pre-treatment serviceable hearing were 0.4 (*p* < 0.01), 0.5 (*p* = 0.05) and 0.6 (*p* = 0.22); for facial nerve impairment, these ORs were 1.1 (*p* = 0.69), 3.5 (*p* = 0.28) and 0.9 (*p* = 0.71), respectively [15]. We can confirm that toxicity to normal tissue and cranial nerves increases for lesions with higher tumor extension grade. Our patients receiving FRT or FPT mostly suffered from large VS with brain stem contact. Thus, these patients were at high risk for cranial nerve toxicity prior to RT. Of all patients without facial nerve impairment prior to RT, 6.9% showed facial nerve toxicity after RT, which was mostly represented as mild or temporary symptoms (switch from HB Class I to Class II). Higher-grade facial nerve toxicity occurred in one patient who received SRS with a maximum dose to the facial nerve of 13.9 Gy. Persson et al reported in a recent review that facial nerve deterioration was present in 3.6% of patients treated with SRS and 11.2% with FRT [16]. The trigeminal nerve deterioration was 6.0% for SRS and 8.4% for FRT. In contrast to our study, patients with NF2 were excluded from the analysis. The increase of cranial nerve toxicity due to higher single-dose exposure and to larger lesions has been reported in the literature [16,17,18,19,20]. Rueß et al., who found lower rates of cranial nerve toxicity investigated only intracanalicular VS with a mean tumor volume of 0.2 ccm whereas our cohort has a mean tumor volume of 1.6 ccm [19]. A previous review reported that trigeminal nerve impairment occurred in 0–10% of patients irradiated with proton radiotherapy with a weighted average of 4% [21]. Altogether, the rates of cranial nerve impairment in our cohort fall within the range commonly reported in comparable literature. Due to the retrospective character of all these data, as well as inherent differences in cohorts and study design, small differences in incidence rates should not be overinterpreted. 

A previous study reported that a Dmax of 5 Gy and above minimum vestibular doses significantly worsened dizziness in patients treated with SRS [22]. In our cohort, no dose constraints were given to the vestibule or the semicircular canals during radiation plan optimization. However, our data indicates that 19.6%-25.8% of our patients reported improvement of vestibular symptoms after RT. In SRS/HFSRT patients, no strong correlation between development of new dizziness, imbalance or gait uncertainty and dose to the labyrinth was observed. Only for FPT patients a correlation between mean median dose to the labyrinth and development of new vestibular symptoms seems possible. Further studies are needed to investigate this supposition.

Hearing deterioration after RT for VS is one of the most relevant factors to influence post-treatment quality of life. Overall, previous data show median hearing preservation rates of 70–80% at 2 years and 55–75% at 5 years after SRT with no statistically difference between SRS and FRT [2,23,24,25]. Our results show higher hearing preservation rates as described above. In particular, patients after SRS had significantly higher hearing preservation rates (hearing deterioration was observed in 3.6% of SRS, 17.8% of FRT and 16.7% of FPT patients), possibly due to smaller tumors in this cohort. The SRS technique offers a more effective and targeted protection of the cochlea. Initial reports of SRS for VS reported low rates of useful hearing preservation (20–50%), and unfavorable preservation of the facial nerve (34–86%) and trigeminal nerve (41–85%) function were seen, especially when higher doses of 16 to 25 Gy were used. Today, doses of SRS have been successfully lowered to reduce nerve toxicity risks while maintaining similar rates of local tumor control [26,27]. We only applied single-dose SRS with a median dose of 12 Gy (preferably to the 80% surrounding isodose). It was previously shown that SRS with a median dose of >13 Gy causes significantly more side effects, in particular hearing deterioration [1,2,9,28,29]. One study showed a trend toward better preservation of hearing after FPT, reducing the total dosage from 54 to 50.4 Gy (RBE) [30]. Moreover, it is known that hearing deterioration increases with time to follow-up. 

The cause of hearing loss after RT for VS is multifactorial. First, patients undergo normal age-related hearing decline. Further, hearing loss and also other cranial nerve deficits can be caused by continuous compression from the tumor on nerves [2]. In addition to treatment-related loss of hearing function, it must be kept in mind that most patients with VS represent an elderly population. Thus, an age-related hearing deterioration (presbycusis) during follow-up after RT can cause a partial misinterpretation of treatment- related issues. 

Only limited data are available on FPT in VS due to the small treatment volumes. FPT offers unique physical properties which enable a dose reduction to normal tissue. Compared to photons, the biological effect is similar, and an RBE of 1.1 is generally used in clinical applications. Considering the reduction of dose to normal tissue in conjunction with a comparable RBE to photons, proton therapy might be the ideal modality to treat patients with benign brain tumors such as VS. In VS the rationale lies not primarily in an increase in local tumor control but in a reduction of side effects, i.e., neurocognitive side effects or secondary malignancies, which are of great importance in patients with benign tumors and a long-life expectancy after treatment. FPT techniques are under strong development and the Heidelberg Ion-Beam Therapy Center (HIT) uses active raster scanning. All patients treated with FPT in our study had larger lesions and therefore received fractionated therapy. No radiosurgery was performed with protons. Proton therapy has physical advantages, with a sharp increase of dose in a well-defined depth (Bragg peak) and a rapid dose falloff beyond that maximum. For treating skull-base tumors, where proton therapy would be expected to have the greatest depth–dose advantage over photon therapy, the lateral penumbra and lateral scattering strongly impacts plan quality relative to photons, due to that lateral scattering protons can be inferior to photons in smaller lesions such as VS Hannover Grade 1–2 [31,32,33]. Further, there is an anatomic variation of density in these areas (e.g., mastoid air cells). These heterogeneities affect the energy distribution in the beam and lead to range uncertainties. Depending on the volume and anatomic conditions, dose distributions with protons can be even inferior or less robust than those of photons [34]. 

It is known that treatment-related side effects significantly influence patients’ post-RT QOL. In our study, we aimed to compare QOL and a detailed symptom questionnaire combined with dosimetric information (e.g., cochlea dose) after proton therapy with photon RT. Data for proton irradiation in patients with VS are rarely available. Further investigations are needed to identify the benefits and risk factors regarding LC, QOL and treatment related side effects after FRT in patients with VS. In line with our results, LC in FPT patients seems to be equally high to those in SRS and FRT [11]. Moreover, we found no statistically significant difference in hearing preservation rate between FPT and photon beam techniques. Only 16.7% of our FPT patients suffered from hearing deterioration; this is in contrast to previous elderly data, which show hearing deterioration in up to 66.6% of patients after FPT [35]. Cranial nerve toxicity seemed to be higher in FPT than in SRS/HFSRT or FRT. Due to the heterogeneous tumor volume in the different treatment groups, higher rates of facial and trigeminal nerve impairment could be caused by selection bias. Of patients receiving FPT, 96.0% had large VS with brain stem contact or compression, which is a commonly known risk factor for cranial nerve impairment [35].

The overall tumor control rates after FPT varied from 85% to 100% [21]. Previous reports of patients treated with hypofractionated proton therapy (26 Gy (RBE) in three fractions) showed local control rates of 98% at 5 years; however, hearing preservation was only 42%, and facial and trigeminal nerve preservation rates of only 90.5 and 93%, respectively [36]. A review on proton therapy for VS reported that the proportion of patients suffering from post-RT hearing loss ranged from 21% to 78%, with an average hearing loss rate of 52% [21]. Average doses to the cochlea were not mentioned in any of the included proton studies. Another study investigated 30 patients treated for 31 vestibular schwannomas with 54 Gy (RBE) protons in 30 fractions for patients with useful hearing and 60 Gy (RBE) in 30–33 fractions for patients without useful hearing. Local control rates were excellent, as no patient demonstrated tumor progression; however, in patients with useful hearing prior to RT, hearing preservation was only 31%. No transient or permanent treatment-related trigeminal or facial nerve dysfunction was reported [37]. 

In a recent study, Koetsier et al. [38] reported on patients with VS treated with protons. The study was retrospective and chart-based, only regarding the investigation of post-irradiation symptoms. Of the total patients studied, 136 received single-fraction proton therapy and 85 patients received FPT. The 5-year local control rate was 96% with a median follow-up of 4.5 years. Progressive post-irradiation speech discrimination score loss occurred in 42% of patients with audiometric follow-up within a year. Facial paresis was found in 5%, severe dizziness in 5%, and trigeminal neuralgia in 5% of patients [38]. Comparable to our study, trigeminal neuralgia occurred more often in patients with larger tumor volumes and in patients that received FPT (*p* = 0.0496). Cochlear mean dose was 43.4 Gy (RBE) for patients treated with FPT [39]. 

Our study has several limitations due to its retrospective nature, as well as due to its design as a cross-sectional study in a population treated over a time period of 10 years. Inherent to this study design, inhomogeneity over time regarding institutional treatment protocols as well as treatment planning and imaging techniques could represent potential confounders that are hard to quantify and address statistically. The available data on FPT are heavily biased, as pre-treatment characteristics significantly in-fluence outcome. In our study, as well as in other proton centers, smaller and therefore less symptomatic lesions are usually treated with single-fraction photon SRS. Patients who received FPT had significantly larger lesions and were more symptomatic. Moreover, the number of patients in the FPT group was much smaller than in the SRS/HFSRT group and FRT group. These conditions harbor an intrinsic selection bias of patients with larger tumors that are subsequently referred for FPT with the aim to minimize collateral brain irradiation. The selection of treatment technique for patients with VS needs to be based on tumor volume, patient characteristics and patient history. In general, cranial nerves affected prior to radiotherapy are more likely prone to further radiation-induced damage. This may indicate a selection bias in previous reports as well as in our own study, possibly resulting in overestimation of cranial nerve injury due to FPT. 

However, no formal randomized study comparing photon RT to FPT has been performed or has assessed patient-related outcomes by self-assessment. It is thus a strength of our study, being to date the only study on self-reported outcome and QOL for FPT patients. Our study furthermore includes a systematic survey with a high questionnaire response rate and a meticulous quantitative classification of symptoms regarding severity. 

Taken together, the few data on proton therapy for VS seem roughly comparable to advanced photons, either with radiosurgery or FRT. Thus currently, no superiority of either treatment method in this indication can be asserted, except possibly for younger patients with FPT, due to the reduction of low-dose exposure of normal tissue leading to a decreased risk for radiation-induced cancers.

## 5. Conclusions

In conclusion, SRS/HFSRT, FRT and FPT are safe and effective methods in the treatment of VS, yielding similar overall clinical and functional outcomes and comparing favorably to previous data. In our cohort, no superiority of either treatment method could be shown for this indication. Selection of treatment technique for patients with VS needs to be based on tumor volume, patient characteristics and patient history. SRS/HFSRT seems to offer the best chances of cranial nerve and specifically hearing preservation. Cranial nerve impairment rates vary, potentially due to selection bias, with larger VS in the FRT and FPT group.

## Figures and Tables

**Table 1:** Patient characteristics by treatment group.

		SRS/HFSRT (*n* = 149)				FRT (*n* = 87)				FPT (*n* = 25)				Total (*n* = 261)			
		Median	IQR	*n*	*n* (%)	Median	IQR	*n*	*n* (%)	Median	IQR	*n*	*n* (%)	Median	IQR	*n*	*n* (%)
**Age [years]**		59	52–72			58	44–70			56	40–66			59	48–71		
Sex	female			74	49.7%			44	50.6%			13	52.0%			131	50.2%
	male			75	50.3%			43	49.4%			12	48.0%			130	49.8%
Localization	left			67	45.0%			43	49.4%			7	28.0%			117	44.8%
	right			79	53.0%			39	44.8%			15	60.0%			133	51.0%
	bilateral			3	2.0%			5	5.7%			3	12.0%			11	4.2%
Genetic	sporadic			140	94.0%			81	93.1%			21	84.0%			242	92.7%
	NF2			1	0.7%			4	4.6%			3	12.0%			8	3.1%
	unknown			8	5.4%			2	2.3%			1	4.0%			11	4.2%
Tumor volume [ccm]		0.65	0.31–1.07			1.59	0.96–2.58			3.93	2.29–6.22			0.80	0.35–1.64		
Surgery	RT as primary treatment			122	81.9%			65	74.7%			18	72.0%			205	78.5%
	Surgery prior to RT			27	18.1%			22	25.3%			7	28.0%			56	21.5%
Time from surgery to RT [months]		52	34–83			33	19–75			12	10–50			43	24–81		

**Table 2:** Differences in tumor volume and tumor extension grade.

		SRS/HFSRT (*n* = 149)				FRT (*n* = 87)				FPT (*n* = 25)				Total (*n* = 261)			
		Median	IQR	*n*	*n* (%)	Median	IQR	*n*	*n* (%)	Median	IQR	*n*	*n* (%)	Median	IQR	*n*	*n* (%)
**Tumor volume [ccm]**		0.65	0.31–1.07			1.59	0.96–2.58			3.93	2.29–6.22			0.80	0.35–1.64		
Tumor extension grade	T1			49	32.9%			17	19.8%			0	0.0%			66	25.4%
	T2			45	30.2%			23	26.7%			1	4.0%			69	26.5%
	T3a			20	13.4%			7	8.1%			0	0.0%			27	10.4%
	T3b			20	13.4%			14	16.3%			4	16.0%			38	14.6%
	T4a			15	10.1%			24	27.9%			20	80.0%			59	22.7%
	T4b			0	0.0%			1	1.2%			0	0.0%			1	0.4%

T1 intracanalicular, T2 intra- and extrameatal, T3a filling cerebellopontine cistern, T3b reaching brainstem, T4a compression of brainstem, T4b compression of the brainstem with dislocation of the 4th ventricle.

**Table 3:** Median doses of organs at risk.

		SRS/HFSRT (*n* = 149)		FRT (*n* = 87)		FPT (*n* = 25)	
Dose [Gy/Gy(RBE)]		1 × 12 Gy (*n* = 120)	3 × 6 Gy HFSRT (*n* = 29)	Total (32 Fractions)	Single Dose	Total (30 Fractions)	Single Dose
**Cochlea**	max.	12.1	17.7	*	*	54.7	1.8
	mean	7.2	12.2	*	*	50.7	1.7
**Labyrinth**	max.	12.7	19.1	*	*	52.7	1.8
	mean	5.6	8.7	*	*	43.6	1.5
**Facial nerve**	max.	14.0	20.0	*	*	54.5	1.8
	mean	5.2	8.4	*	*	43.5	1.5
**Right optic nerve**	max.	0.9	1.9	0.6	0.0	0.6	0.0
	mean	0.1	0.3	0.2	0.0	0.0	0.0
**Left optic nerve**	max.	1.0	1.2	0.4	0.0	0.2	0.0
	mean	0.1	0.2	0.1	0.0	0.0	0.0
**Optic chiasm**	max.	1.3	2.2	1.0	0.0	5.4	0.2
	mean	0.4	0.9	0.4	0.0	0.7	0.0
**Brain stem**	max.	4.8	17.6	51.6	1.6	53.8	1.8
	mean	0.6	1.6	6.2	0.2	11.4	0.4

* Dosimetric information was not available.

## Data Availability

Data available on request due to data sharing restrictions. The data presented in this study are available on request from the corresponding author.
